# Long COVID and recovery from Long COVID: quality of life impairments and subjective cognitive decline at a median of 2 years after initial infection

**DOI:** 10.1186/s12879-024-10158-w

**Published:** 2024-11-05

**Authors:** Warren Szewczyk, Annette L. Fitzpatrick, Herve Fossou, Nicole L. Gentile, Nona Sotoodehnia, Surabhi B. Vora, T. Eoin West, Jeanne Bertolli, Jennifer R. Cope, Jin-Mann S. Lin, Elizabeth R. Unger, Quan M. Vu

**Affiliations:** 1 Department of Epidemiology, School of Public Health, University of Washington, Seattle, WA USA; 2 Department of Family Medicine, School of Medicine, University of Washington, Seattle, WA USA; 3 Department of Medicine, Cardiovascular Health Research Unit, University of Washington, Seattle, WA USA; 4 Division of Infectious Diseases, Department of Pediatrics, Seattle Children’s Hospital, Seattle, WA USA; 5 Division of Pulmonary, Critical Care and Sleep Medicine, Department of Medicine, University of Washington, Seattle, WA USA; 6 Centers for Disease Control and Prevention, Atlanta, GA USA

**Keywords:** Long COVID, Post-COVID condition, Quality of life, Cognition, Recovery, Subjective cognitive decline, Post-acute sequelae

## Abstract

**Background:**

Recovery from SARS CoV-2 infection is expected within 3 months. Long COVID occurs after SARS-CoV-2 when symptoms are present for more than 3 months that are continuous, relapsing and remitting, or progressive. Better understanding of Long COVID illness trajectories could strengthen patient care and support.

**Methods:**

We characterized functional impairments, quality of life (QoL), and cognition among patients who recovered from SARS-CoV-2 infection within 3 months (without Long COVID), after 3 months (Recovered Long COVID), or remained symptomatic (Long COVID). Among 7305 patients identified with previous SARS-CoV-2 infection between March 2020 and December 2021, confirmed in the medical record with laboratory test or physician diagnosis, 435 (6%) completed a single self-administered survey between March 2022 and September 2022. Multi-domain QoL and cognitive concerns were evaluated using PROMIS-29 and the Cognitive Change Index-12.

**Results:**

Nearly half the participants (47.7%) were surveyed more than 2 years from initial infection (median = 23.3 months; IQR = 18.6, 26.7) and 86.7% were surveyed more than 1 year from infection. A significantly greater proportion of the Long COVID (*n* = 215) group, (Current and Recovered combined), had moderate-to-severe impairment in all health domains assessed compared to those Without Long COVID (*n* = 220; all *p* < 0.05). The Recovered Long COVID group (*n* = 34) had significantly lower prevalence of fatigue, pain, depression, and physical and social function impairment compared to those with Current Long COVID (*n* = 181; all *p* < 0.05). However, compared to patients Without Long COVID, the Recovered Long COVID group had greater prevalences of fatigue, pain (*p* ≤ 0.06) and subjective cognitive decline (61.8% vs 29.1%; *p* < 0.01). Multivariate relative risk (RR) regression indicated Long COVID risk was greater for older age groups (RR range 1.46–1.52; all *p* ≤ 0.05), those without a bachelor’s degree (RR = 1.33; 95% CI = 1.03–1.71; *p* = 0.03), and those with 3 or more comorbidities prior to SARS-CoV-2 infection (RR = 1.45; 95% CI = 1.11–1.90; *p* < 0.01).

**Conclusions:**

Long COVID is associated with long-term subjective cognitive decline and diminished quality of life. Clinically significant cognitive complaints, fatigue, and pain were present even in those who reported they had recovered from Long COVID. These findings have implications for the sustainability of participation in work, education, and social activities.

**Supplementary Information:**

The online version contains supplementary material available at 10.1186/s12879-024-10158-w.

## Introduction & objective

Evidence throughout the novel coronavirus-19 (COVID-19) pandemic has demonstrated that a significant proportion of those infected with SARS-CoV-2 (the virus that causes COVID-19) experience a “continuation or development of new symptoms” more than 3 months after initial infection, termed a “post COVID-19 condition” (PCC or “Long COVID”) by the World Health Organization (WHO) [1–6]. Fatigue, post-exertional malaise, unrefreshing sleep, pain, and neurocognitive symptoms have been consistently associated with Long COVID even after mild acute infections [3, 4, 6–13], drawing comparisons to myalgic encephalomyelitis/chronic fatigue syndrome (ME/CFS) [3]. Long COVID is recognized to be an infection-associated chronic condition whose presentations include a wide variety of symptoms and diagnosable conditions, complicating studies of illness trajectory and the clinical management of patients [14].

The association of Long COVID with impairments in both quality of life (QoL) and cognition are persistent and significant [4, 7–10, 12, 15–18]. The data on symptoms more than 2 years after infection are limited and confined largely to research involving hospitalized patients and medical record studies [12, 15, 17, 19]. As such, the long term relationship between mild-to-moderate COVID-19 illness and QoL, which is not routinely captured in the medical record, is unclear. Impacts on memory and attention have also been associated with Long COVID at follow-up intervals of more than 6 months [4, 8, 20, 21]. Though changes in cognition can be difficult to assess without repeated neuropsychological measurement, a body of literature has shown that subjective perception is a meaningful measure of cognitive changes [22–25]. Self-reported cognitive complaints have clinical relevance, as objective testing is often indicated after they arise, and they have been associated with future cognitive impairment in older adults [24, 26, 27]. Cognition is a key determinant of daily functioning and is associated with quality of life [28, 29], and thus represents an important domain to measure when assessing functional impairment from Long COVID.

While the 6 to 12 months following COVID-19 illness are currently the best characterized, more recent studies that include follow-up of 24 months or more continue to identify significant health impairments [7, 15, 30, 31]. Studies utilizing participant self-report with long follow-up are crucial for understanding the medium- and long-term trajectories of Long COVID and the related impacts on QoL and day-to-day-functioning. Better characterizing functional impairment among those with Long COVID is an important scientific goal given that data from the Centers for Disease Control and Prevention (CDC) indicate roughly a quarter of U.S. adults with Long COVID have significant limitations to regular activities [32].

In this study, our objectives were to characterize long-term functional impairments, cognition, and quality of life among those who self-reported having Long COVID, including comparing patients with current Long COVID to those who recovered from Long COVID, and participants without Long COVID. We also investigated associations of demographic and clinical factors with Long COVID. We used validated measures to characterize current QoL and subjective cognitive decline at a median of approximately 2 years after initial infection in a sample of patients with medical record confirmed COVID-19, a majority of whom were not hospitalized during the acute illness.

## Methods

### Participant selection

**Fig.1:**
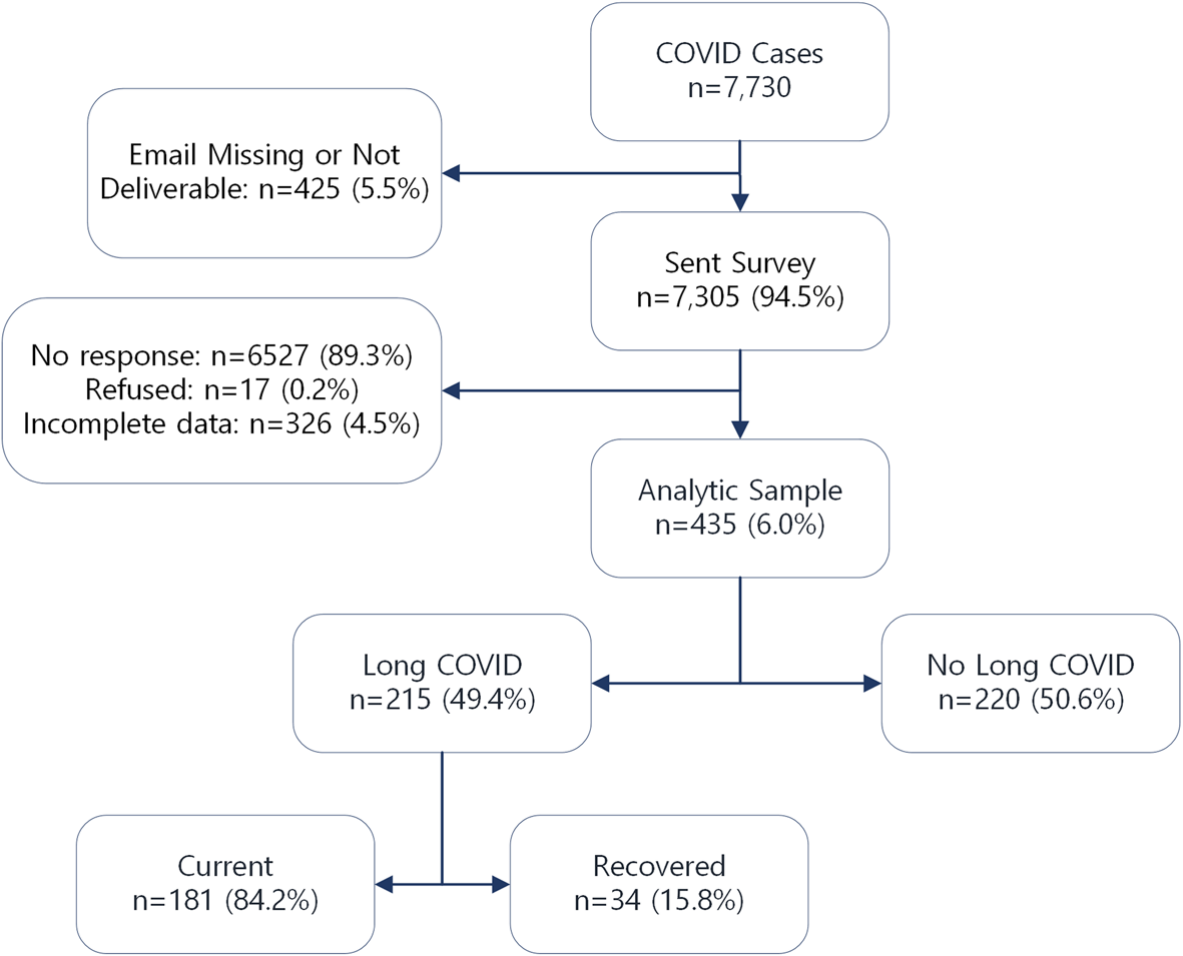
Participant flow diagram. Flow diagram outlining the creation of the analytic sample for this study (*N* = 435) and the grouping of Long COVID (*n* = 215) vs Without Long COVID (*n* = 220) within the sample. Percentages were computed by dividing each n by the number of individuals in the preceding box

We collected all inpatient and outpatient medical records within an urban, university-affiliated medical system as part of the Centers for Disease Control and Prevention’s (CDC) COVID-RELIEF (Research on COVID-19 Long Term Effects) project. Adults with 1) indication of SARS-CoV-2 infection – that is, a positive SARS-CoV-2 polymerase chain reaction (PCR) test result or a clinical diagnosis of COVID-19 (*n* = 120 diagnosed by PCR, 27.5%) – between March 2020 and December 2021 and 2) a patient ZIP code within the state were included in the cohort. A clinical diagnosis of COVID-19 was defined as an International Classification of Diseases, 10th Revision, Clinical Modification (ICD-10-CM) diagnostic code of B97.29 (“Other coronavirus as the cause of diseases classified elsewhere”), and/or U07.1 (“COVID-19”; see Supplemental Table S3 for comparison of patients by identification method). All those identified were selected for follow-up surveys and patients with an available email address were emailed an invitation to join a COVID-19 research project. All participants who provided data on their COVID-19 recovery and at least partially completed the outcome measures and demographics were included in the analysis (*N* = 435; response rate = 6.0%; Fig. 1). Surveys were completed between March 2022 and September 2022, at least 4 months after initial infection for all participants.

### Study measures

Participants completed surveys remotely via a secure instance of Research Electronic Data Capture (REDCap; https://redcap.iths.org). The surveys collected basic sociodemographics (all categorical variables self-classified) and information about acute COVID-19 illness, including hospitalization, pre-COVID comorbidities, the number of symptoms experienced out of 11 core symptoms, recovery status (asked as “Have you returned to your pre-COVID baseline health?”), and time to recovery, if applicable (see Supplemental Information) [33]. The Long COVID group was defined as all participants who were not recovered at time of survey (Current Long COVID) or who experienced recovery or remission more than 3 months after initial infection (Recovered Long COVID). The comparison group (Without Long COVID) were those who reported recovery to pre-COVID baseline health in ≤ 3 months.

Participants completed the 29-item Patient Reported Outcomes Measures Information System v2.0 (PROMIS-29) survey. The survey has 7 subscales that assess the current status of the following health domains that impact quality of life: physical function, anxiety, depression, fatigue, sleep disturbance, social function (i.e., ability to participate in social roles and activities), and pain interference [34, 35]. Each subscale is composed of four questions each (Likert ratings from 1 – 5; individual subscale score range, 4 – 20). For all scales but the social and physical function scales, higher scores indicate greater impairment. The seven subscales demonstrate high marginal reliability, with reliability coefficients ranging from 0.73 to 0.91 [34].

To measure subjective cognitive decline, participants completed the 12-item Cognitive Change Index (CCI-12) [36], modified to ask for comparisons of current memory to pre-COVID-19 baseline. The CCI-12 responses are captured on a 1–5 Likert scale and summed (range: 12 – 60), with greater scores indicating more severe perceived changes [25, 36]. An additional item, not included in the sum total, assessed participants’ concern about cognitive changes on a 1 – 5 Likert scale. The psychometric properties of the 12-item CCI have not been published, but the 20-item Cognitive Change Index (CCI-20) from which the CCI-12 is drawn has high internal consistency (Cronbach’s *α* = 0.98), and the CCI-12 shows strong correlations with the other two subscales of the CCI-20 (Pearson’s *r* = 0.81 with the Executive Function subscale and *r* = 0.71 with the Language subscale) [36].

### Statistical analysis

Descriptive statistics were calculated for each group and compared with *t*-tests for continuous variables and Chi-square tests for categorical variables. Raw PROMIS totals were converted to normalized T-scores for each domain and participants were classified as having moderate or severe domain-specific impairment based on clinically validated thresholds [35, 37, 38]. The CCI-12 scores were summed, with scores ≥ 20 indicating meaningful cognitive decline [22, 25, 39]. A threshold of one standard deviation (sample-derived) above the validated clinical threshold (i.e., > 32) was chosen to explore severity differences by group. Participants were classified as having cognitive concerns if they reported being at least “Slightly Concerned” about memory changes since COVID-19. For each domain, we compared the proportion of moderate-to-severe impairment across groups using Chi-square tests or Fisher’s exact test and a significance level of α = 0.05. Bonferroni correction was applied to comparisons of moderate or above impairment, but not severe, due to small sample size.

To model the relative risk (RR) of social and health factors associated with Long COVID, we fit a quasi-Poisson regression with a robust error variance using Long COVID (comprised of Current Long COVID and Recovered Long COVID) as a binary outcome [40, 41]. All independent variables were selected a priori*,* and unadjusted bivariate RRs were estimated for each variable. Variables with an unadjusted association (*p* < 0.10) were included in the multivariable model.

### Missing data & sensitivity analyses

The proportions of observations with missing data were generally low (< 1% for 40.4% of variables with at least 1 missing observation, see Supplemental Table S1). Missing PROMIS and CCI-12 values were mean-imputed. Sensitivity analyses were conducted by inputting extreme values (i.e., all lowest values for Long COVID group and highest values for Without Long COVID comparison, see Supplemental Information). For each categorical variable in the regression model, missing values were coded as a separate category. Groups with n < 5 were dropped from the regression model, as preliminary modeling indicated high leverage from these groups. Hospitalization was not included in the model due to a high proportion of missingness (13.1%). An additional sensitivity analysis was conducted to assess the impact of COVID-19 identification method (PCR vs ICD-10 code) on the model results. Lastly, we assessed whether time from diagnosis was relevant to functional impairment in the Current Long COVID group by comparing the prevalence of moderate-to-severe impairment in those who were surveyed 2 or more years since index diagnosis to those surveyed less than 2 years since index diagnosis (see Supplemental Information).

Data were analyzed with R v4.3.1. The study was approved by the University of Washington Institutional Review Board (IRB) and by the CDC in accordance with CDC policy and applicable regulations. Written informed consent was obtained from all survey respondents; a waiver of informed consent was granted by the University of Washington IRB for de-identified data collection from the health record and survey recruitment messages.

## Results

### Descriptive statistics

A total of 435 participants provided sufficient survey data for analysis among the 7305 patients invited to complete surveys (response rate = 6.0%). Their median time since initial SARS-CoV-2 infection was 23.3 months (Interquartile Range [IQR] = 18.6, 26.7 months; range = 3.9, 30.0 months). Slightly more than half of the participants recovered ≤ 3 months after the acute illness (Without Long COVID, 220/435, 50.6%). Of the Long COVID group (215/435, 49.4%), most were Current Long COVID (181/215, 84.2%) and a minority were Recovered Long COVID (34/215, 15.8%). The median recovery time, as measured from the index date of diagnosis, for the Without Long COVID group was 11.9 days (IQR = 4.9, 22.2 days; range = 0, 90 days) while for the Recovered Long COVID, median recovery time was 240.0 days (7.89 months; IQR = 5.92, 13.6 months; range = 3.9, 26.2 months). The median time from index diagnosis date to survey for the Current Long COVID group was 24.1 months (IQR = 19.7, 26.8 months; range = 4.96, 30.0 months).

Approximately 61.1% of the overall sample was age 50 or over (mean age = 53.4, SD = 14.9), and the age distributions did not differ significantly between the Long COVID and Without Long COVID groups (*p* = 0.19) (Table 1). When compared to the group Without Long COVID, the Long COVID group had a greater percentage of women (65.6% to 55.9%, *p* < 0.05) and people without a bachelor’s degree (39.5% to 25.9%, *p* < 0.01). A greater proportion of the Long COVID group had been hospitalized during acute illness (7.0% to 1.8%, *p* < 0.01), had a respiratory comorbidity prior to COVID-19 (17.2% to 10.0%, *p* < 0.05), and had 3 or more comorbidities (8.8% to 3.2%, *p* < 0.05). The group Without Long COVID reported experiencing approximately 2 fewer symptoms during acute illness than the Long COVID group (*p* < 0.01) and 1 fewer current symptom (*p* < 0.01). The Recovered Long COVID group was not significantly different from the Current Long COVID group on these descriptive variables (See Supplemental Table 2).

**Table 1:** Descriptive statistics comparing Long COVID (> 3 month recovery from SARS-CoV-2 infection) to without Long COVID (≤ 3 month recovery) assessed cross-sectionally at a median of 2 years after initial infection

	Total (*N* = 435)	Long COVID (*N* = 215)	Without Long COVID (*N* = 220)	*p*-value^a^
**Age**				0.19
20–34	61 (14.0%)	24 (11.2%)	37 (16.8%)	
35–49	105 (24.1%)	53 (24.7%)	52 (23.6%)	
50–64	148 (34.0%)	81 (37.7%)	67 (30.5%)	
65 +	118 (27.1%)	54 (25.1%)	64 (29.1%)	
**Gender**				0.03^b^
Male	166 (38.2%)	71 (33.0%)	95 (43.2%)	
Female	264 (60.7%)	141 (65.6%)	123 (55.9%)	
Other	3 (0.7%)	2 (0.9%)	1 (0.5%)	
**Education**				< 0.01
Advanced degree	123 (28.3%)	48 (22.3%)	75 (34.1%)	
Bachelor’s degree	166 (38.2%)	79 (36.7%)	87 (39.5%)	
No bachelor’s degree	142 (32.6%)	85 (39.5%)	57 (25.9%)	
**Race**				0.60
White	340 (78.2%)	163 (75.8%)	177 (80.5%)	
Asian	27 (6.2%)	14 (6.5%)	13 (5.9%)	
Black	27 (6.2%)	13 (6.0%)	14 (6.4%)	
American Indian-Alaska Native	8 (1.8%)	6 (2.8%)	2 (0.9%)	
Other	20 (4.6%)	12 (5.6%)	8 (3.6%)	
**Hispanic ethnicity**				0.40
Not Hispanic	385 (88.5%)	187 (87.0%)	198 (90.0%)	
Hispanic	39 (9.0%)	22 (10.2%)	17 (7.7%)	
**COVID hospitalization**	19 (4.4%)	15 (7.0%)	4 (1.8%)	< 0.01
Missing	57 (13.1%)	39 (18.1%)	18 (8.2%)	
**Pre-infection comorbidities**				
Cardiovascular (Non-Hypertension)	39 (9.0%)	25 (11.6%)	14 (6.4%)	0.05
Hypertension	86 (19.8%)	47 (21.9%)	39 (17.7%)	0.30
Diabetes	30 (6.9%)	19 (8.8%)	11 (5.0%)	0.10
Respiratory	59 (13.6%)	37 (17.2%)	22 (10.0%)	0.03
Clotting	18 (4.1%)	11 (5.1%)	7 (3.2%)	0.30
Auto-immune	41 (9.4%)	22 (10.2%)	19 (8.6%)	0.60
Pre-infection comorbidity count				0.04
0	253 (58.2%)	117 (54.4%)	136 (61.8%)	
1	106 (24.4%)	50 (23.3%)	56 (25.5%)	
2	50 (11.5%)	29 (13.5%)	21 (9.5%)	
3 or more	26 (6.0%)	19 (8.8%)	7 (3.2%)	
**Acute symptom total;** Mean (SD)^c^	5.34 (2.83)	6.40 (2.66)	4.32 (2.60)	< 0.01
**Current symptom total;** Mean (SD)	1.04 (1.73)	1.76 (2.07)	0.34 (0.85)	< 0.01
**Recovery time (mos);** Median (IQR)	0.46 (0.20, 1.973)	7.89^d^ (5.92, 13.6)	0.39 (0.16, 0.73)	< 0.01
**Time since infection (mos);** Median (IQR)	23.3 (18.6, 26.7)	24.1 (19.3, 26.8)	22.5 (17.6, 26.7)	< 0.01

^a^Significance testing conducted with *t*-tests for continuous variables and Chi-squared tests for categorical

^b^Results shown for pairwise comparison of Males and Females

^c^Symptom totals (both during the acute phase of COVID-19 and current) are counted out of 11 core symptoms defined by the Centers for Disease Control and Prevention: fever or chills, cough, shortness of breath, fatigue, muscle or body aches, headache, new loss of taste or smell, sore throat, congestion or runny nose, nausea or vomiting, and diarrhea

^d^Represents the *n* = 34 in the Long COVID group who were recovered at time of survey

**Table 2:** Prevalence of functional impairment across 8 health domains comparing Long COVID (Current and recovered) to without Long COVID

	Total (*N* = 435)	Long COVID (*N* = 215)	Without Long COVID (*N* = 220)	Prevalence Ratio (95% CI)	*p*-value
**Physical function**	96 (22.1%)	77 (35.8%)	19 (8.6%)	4.17 (2.62, 6.64)	< 0.01
Severe	17 (17.7%)	14 (18.2%)	3 (15.8%)	1.15 (0.37, 3.61)	1.0
**Anxiety**	81 (18.6%)	59 (27.4%)	22 (10.0%)	2.76 (1.75, 4.33)	< 0.01
Severe	24 (29.6%)	22 (37.3%)	2 (9.1%)	4.10 (1.05, 16.0)	0.01
**Depression**	54 (12.4%)	38 (17.7%)	16 (7.3%)	2.44 (1.40, 4.24)	< 0.01
Severe	7 (13.0%)	7 (18.4%)	0	–	0.09
**Fatigue**	106 (24.4%)	88 (40.9%)	18 (8.2%)	4.98 (3.11, 7.97)	< 0.01
Severe	24 (22.6%)	24 (27.3%)	0	–	0.01
**Sleep disturbance**	54 (12.4%)	42 (19.5%)	12 (5.5%)	3.58 (1.94, 6.61)	< 0.01
Severe	8 (14.8%)	8 (19.0%)	0	–	0.18
**Social function**	55 (12.6%)	49 (22.8%)	6 (2.7%)	8.36 (3.66, 19.1)	< 0.01
Severe	16 (29.1%)	16 (32.7%)	0	–	0.17
**Pain**	92 (21.1%)	77 (35.8%)	15 (6.8%)	5.30 (3.15, 8.92)	< 0.01
Severe	14 (15.2%)	11 (14.3%)	3 (20.0%)	0.71 (0.23, 2.26)	0.7
**Cognitive decline**	219 (50.3%)	155 (72.1%)	64 (29.1%)	2.49 (1.99, 3.11)	< 0.01
Severe	101 (46.1%)	86 (55.5%)	15 (23.4%)	2.37 (1.49, 3.77)	< 0.01
**Cognitive concern**	246 (56.6%)	165 (76.7%)	81 (36.8%)	2.10 (1.75, 2.54)	< 0.01

Percentages in the “Severe” rows represent the proportion of severe cases among those exceeding thresholds for moderate-to-severe impairment in each domain. All domains except Cognitive Decline and Cognitive Concern assessed with the Patient Reported Outcomes Measurement Information System (PROMIS)-29 v2.0 subscales with raw totals converted to T-scores. T-scores ≥ 60 indicated moderate impairment, ≥ 70 indicated severe for all domains except Physical Function and Social Function, for which T-scores ≤ 40 indicated moderate impairment, ≤ 30 indicated severe. Cognitive domains assessed with the Cognitive Change Index-12. Sum totals ≥ 20 indicated moderate Cognitive Decline, ≥ 33 indicated severe. Cognitive Concern assessed with 1-item and defined as “Slight Concern” or above. Significance testing conducted with Chi-squared tests or Fisher’s Exact test for expected cell counts ≤ 5. Bonferroni correction applied to moderate-to-severe impairment comparisons but not severe comparisons due to small sample size. Prevalence ratios not calculable for domains where no severe cases were observed in the Without Long COVID group

### Current impairments

The Long COVID group (Current and Recovered combined) showed significantly increased prevalence of current functional impairment in every domain assessed, compared with the group Without Long COVID (all *p* < 0.01; Table 2). Subjective cognitive decline was prevalent in both the Long COVID group and the Without Long COVID comparison groups (72.1% and 29.1%, respectively). After cognitive decline, fatigue (40.9%), pain (35.8%), physical function impairment (35.8%), and anxiety (27.4%) were most prevalent in the Long COVID group, whereas in the group Without Long COVID, the next most prevalent impairments after cognitive decline were anxiety (10.0%), physical function (8.7%), fatigue (8.2%), and depression (7.3%). Social function impairment was more than 8 times as prevalent in the Long COVID group (22.8%) than in those Without Long COVID (2.7%).

Among those with impairment of at least moderate severity in each domain, a greater proportion had severe fatigue (27.3%), anxiety (37.3%), and cognitive decline (55.5%) in the Long COVID group compared with those Without Long COVID (0%, 9.1%, and 23.4%, respectively; Table 2). While pain interference and physical function impairment were more prevalent overall in the Long COVID group (both *p* < 0.01), severity in these domains was not different between the two groups.

**Fig. 2:**
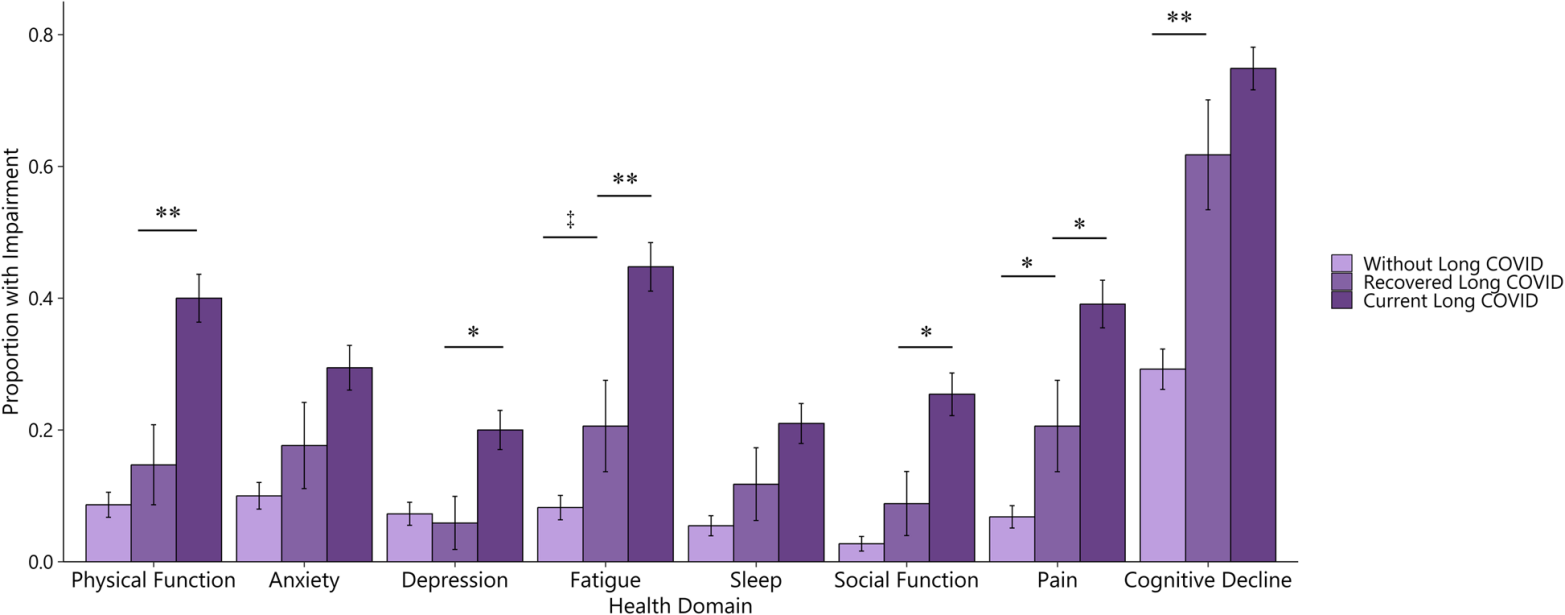
Prevalence of impairment across 8 health domains for Without Long COVID-19 (*n* = 220), Current Long COVID (*n* = 181), and Recovered Long COVID (*n* = 34) Groups. Pairwise comparisons of proportions were performed with Chi-squared or Fisher’s exact tests. The Current Long COVID group was significantly different from the Without Long COVID group in every domain (significance testing not shown). * *p*  < 0.05 ** *p*  < 0.01 ‡ *p*  < 0.10

The Recovered Long COVID group (*n* = 34) showed an elevated prevalence of current moderate-to-severe impairment in multiple domains compared to those Without Long COVID (Fig. 2). A majority of the Recovered Long COVID group reported cognitive decline since COVID-19 (61.8%). The next most prevalent impairments were fatigue and pain (20.6% each), anxiety (17.6%), and physical function (14.7%). Cognitive decline and pain were significantly more prevalent in the Recovered Long COVID group compared with those Without Long COVID (both *p* < 0.05). We also observed an elevated prevalence of fatigue that was not statistically significant (*p* = 0.06). A greater proportion of the Recovered Long COVID group (67.6%) reported concern about memory changes since COVID-19 compared to those Without Long COVID (36.8%; *p* < 0.01; results not shown). Compared with the Current Long COVID group, the Recovered Long COVID group showed decreased prevalence of fatigue, depression, pain, and both social and physical function impairment (all *p* < 0.05). We did not find evidence that those with Current Long COVID who were surveyed 2 or more years since index diagnosis had significantly different prevalences of health impairment compared to Current Long COVID surveyed less than 2 years since index diagnosis (see Supplemental Information).

### Long COVID risk factor

Results from the multivariable regression indicated that age, education, pre-COVID-19 comorbidities, and the number of acute phase symptoms were all associated with Long COVID (i.e., Current Long COVID and Recovered Long COVID; Table 3). Compared with those with an advanced degree, patients without a bachelor’s degree had approximately 33% greater adjusted risk of Long COVID (adjusted RR [aRR] = 1.33; 95% CI = 1.03, 1.71; *p* = 0.03). Compared with those 20–34 years old, older age groups each had roughly the same magnitude of increased risk, ranging from a 47% greater adjusted risk for ages 35 – 49 (aRR = 1.47; 95% CI = 1.01, 2.13; *p* < 0.05) to a 52% greater risk for ages 50 – 64 (aRR = 1.52; 95% CI = 1.06, 2.18; *p* < 0.05). Women had a 23% greater risk of Long COVID than men (aRR = 1.23; 95% CI = 1.00, 1.51; *p* = 0.06). Patients with 3 or more comorbidities before COVID-19 had a 45% elevated risk of Long COVID compared to those with no comorbidities (aRR = 1.45, 95% CI = 1.11, 1.90; *p* < 0.01) and each additional symptom during the acute infection was associated with 14% greater risk in the full multivariable model (aRR = 1.14; 95% CI = 1.10, 1.18; *p* < 0.01). In an unadjusted model, people who self-classified as American Indian or Alaska Native showed increased risk of Long COVID (RR = 1.56, 95% CI = 1.03, 2.37), but the effect was attenuated and non-significant in the multivariable model. No other significant effects were seen for race or ethnicity in unadjusted or adjusted comparisons.

**Table 3:** Bivariate and multivariable relative risk regression model of Long COVID (Current and Recovered)

	Unadjusted models		Multivariable model^a^	
	RR (95% CI)	p-value	Adj. RR (95% CI)	*p*-value
**Age**				
20 – 34	Reference	–	Reference	–
35 – 49	1.28 (0.89, 1.85)	0.18	1.47 (1.01, 2.13)	0.05
50 – 64	1.39 (0.99, 1.96)	0.06	1.52 (1.06, 2.18)	0.03
65 +	1.16 (0.81, 1.68)	0.42	1.48 (1.01, 2.18)	0.05
**Gender**				
Male	Reference	–	Reference	–
Female	1.25 (1.01, 1.53)	0.03	1.23 (1.00, 1.51)	0.06
**Education**				
Advanced Degree	Reference	–	Reference	–
Bachelor’s Degree	1.22 (0.93, 1.60)	0.17	1.12 (0.87, 1.46)	0.38
No bachelor’s Degree	1.53 (1.18, 1.99)	< 0.01	1.33 (1.03, 1.71)	0.03
**Race**				
White	Reference	–	Reference	–
Asian	1.08 (0.74, 1.59)	0.66	1.12 (0.75, 1.67)	0.59
Black	1.00 (0.67, 1.51)	0.96	0.90 (0.62, 1.29)	0.57
American Indian/Alaska Native	1.56 (1.03, 2.37)	0.03	1.09 (0.67, 1.78)	0.73
Other	1.25 (0.67, 1.88)	0.23	1.06 (0.66, 1.71)	0.81
**Hispanic ethnicity**				
Not Hispanic	Reference	–	–	–
Hispanic	1.16 (0.86, 1.56)	0.32	–	–
**Pre-infection comorbidities**				
No comorbidities	Reference	–	Reference	–
1	1.02 (0.80, 1.30)	0.87	0.93 (0.74, 1.18)	0.56
2	1.25 (0.96, 1.64)	0.12	1.15 (0.89, 1.50)	0.29
3 or more	1.58 (1.21, 2.07)	< 0.01	1.45 (1.11, 1.90)	< 0.01
**Total number of acute symptoms**	1.15 (1.11, 1.19)	< 0.01	1.14 (1.10, 1.18)	< 0.01

*RR* Relative Risk, *CI* Confidence Interval

^a^A total of 424 participants were included in the final model. “Missing” categories for age (*n* = 3), gender (*n* = 2), and education (*n* = 4), and the Other category for gender (*n* = 3) were dropped from the analysis due to group size of < 5. Not displayed: “Missing” categories for race (*n* = 12) and ethnicity (*n* = 10)

## Discussion

In this study, patients with Long COVID were compared with those who reported recovering from symptoms within 3 months of the acute phase of COVID-19. This study’s long follow-up time, medical-record confirmed SARS-CoV-2 infection date, and use of population-normed, clinically validated survey measures allow characterization of the long-term functional impact of Long COVID. The results suggest a range of functional impairments across different QoL domains are associated with a lack of recovery from COVID-19 within 3 months, and these impairments can be detected at a median of 2 years after initial infection. Among the Long COVID group, a minority (15.8%) reported being recovered or in remission from COVID-19 at time of survey, with a median reported recovery time of 7.9 months (Recovered Long COVID). While acute infection severity (approximated with total symptom count) was found to be associated with Long COVID, > 80% of this sample were not hospitalized during the acute phase of infection, underscoring the potential for concerning long-term sequelae even with mild-to-moderately severe infection. Beyond physical complaints, including fatigue and pain, the Long COVID group showed significantly decreased social function and increased anxiety, depression, and cognitive decline.

Our findings indicate that recovery or remission from Long COVID occurs in some of the mental and physical health domains assessed. The prevalence of moderate-to-severe fatigue, pain, depression, and both physical and social functional impairment were all significantly lower in the Recovered Long COVID group than in the Current Long COVID group. Of concern, however, the prevalence of cognitive decline, fatigue, and pain remained elevated in the Recovered Long COVID group compared with the Without Long COVID group. This may indicate that some domains of Long COVID-related impairment are more amenable to recovery than others. Alternatively, those reporting recovery from Long COVID may not attribute their current functional impairment to COVID-19, or their functional impairments could have preceded COVID-19. Because Long COVID symptoms can remit and relapse over time, those reporting recovery at time of survey after having Long COVID may have a future return of symptoms or increases in impairment.

Investigating the longitudinal trajectories of patient-reported outcomes in different health domains and their interactions could help advance the understanding of Long COVID and support recovery. Those who recovered may have utilized therapeutics to aid recovery. Studies identifying effective therapeutic approaches to Long COVID recovery are needed and should explore techniques employed by patients. It should be noted that the wording of the recovery question in this study did not probe for remission of symptoms and may have overrepresented people with continuous symptoms from time of diagnosis until survey and underrepresented those with delayed onset of Long COVID. Those with continuous symptoms may have greater Long COVID severity, which could help explain the high degree of impairment observed in this study. Another important caveat is that those reporting recovery from Long COVID at time of survey may be experiencing a temporary remission, and thus cannot be considered permanently recovered from Long COVID. Further work is needed to determine the probability of relapse after subjective recovery from Long COVID, especially after long follow up time from initial infection.

### Quality of life and cognitive decline

The QoL impairments detected in this study are similar to pooled prevalences calculated from 12 published studies of Long COVID in which all participants were hospitalized [17]. The impact on social function associated with Long COVID was prominent – 33% had severe impairment – and is consistent with qualitative work that identified Long COVID symptomatology as a barrier to social well-being [42]. Similarly, the prevalence of severe anxiety was 37% among Long COVID patients with anxiety, and among the overall Long COVID group, more than 10% reported severe anxiety. While moderate-to-severe pain and physical function impairment were more prevalent in the Long COVID group than among those Without Long COVID, the prevalences of severe impairment among those with at least moderate impairment in these domains were not significantly different. These findings underscore the importance of cognitive, mental health and social function impairments in Long COVID, which may increase in prevalence over time relative to other sequelae [7, 12, 43]. While Long COVID is not a mental illness, clinical care for Long COVID patients requires attention to mental health along with treatment for physical symptoms, given the high prevalence of severe anxiety and limitations to social functioning in the Long COVID group.

The high proportion of cognitive decline, even among patients Without Long COVID, could indicate underrecognized long-term cognitive impacts of COVID-19. The proportion with subjective cognitive decline in this sample is notably higher than population-based and meta-analytic prevalence estimates from data collected before the COVID-19 pandemic, but different methodologies among studies prevent a clear comparison [44, 45]. Troublingly, 29% of the Without Long COVID group in the current study showed evidence of cognitive decline since COVID-19 illness, and 36% indicated concern about their memory. This aligns with evidence demonstrating that people with mild past COVID-19 illness, reporting no current symptoms, showed lower cognitive performance than expected on neuropsychological testing [46]. Our study did not have a group with no evidence of SARS-CoV-2 infection for comparison with the Without Long COVID group. Published evidence suggests the prevalence we report may be elevated for a sample with a mean age of 54, given that a global pooled analysis found an age- and gender-standardized prevalence of subjective cognitive decline to be 33% – in a sample with a mean age nearly 20 years older [45]. The CCI-12 cut-offs used to operationalize cognitive decline in this study have not undergone extensive validation and could overestimate the true prevalence of cognitive decline. However, 93% of this sample with a CCI-12 score of ≥ 20 reported a concern about memory changes, whereas only 17% of those with a score of < 20 reported concern, suggesting the chosen threshold measures differences that are relevant to patients.

Early report of perceived cognitive deficit has been associated with later Long COVID [21], and neurocognitive symptoms of Long COVID have been associated with decreased likelihood of working full-time and have been qualitatively connected to social isolation and work discrimination [42, 47]. A large medical record study of post-acute sequelae at 2 years post-infection found a greater 2-year cumulative disability burden from both neurological and mental health symptoms than from fatigue, pulmonary symptoms, or musculoskeletal symptoms [19]. Since subjective cognitive decline has been associated with more severe future cognitive impairment in patients without Long COVID [26], the reported memory concerns suggest the clinical importance of carefully monitoring the cognitive function of those who do not recover from COVID-19 within 3 months. Healthcare providers should be aware of the potential for longer term cognitive impacts after COVID-19 illness, including for those who report mild symptoms, a short recovery time, or subjective recovery after prolonged symptoms. This issue may be especially pronounced for patients who had COVID-19 during the more severe early waves of the pandemic – as nearly 80% of the sample in the current study did – or who experienced cognitive symptoms during the acute infection. Future studies examining the longitudinal correlates of cognitive decline in the context of Long COVID could be valuable, and further investigation as to how cognitive decline impacts daily functioning for those with Long COVID is warranted.

### Long COVID risk factors

While ages 35 and above were associated with increased Long COVID risk in this study compared to those ages 20 – 34, the risk increase was roughly equivalent among each older age group which is generally consistent with CDC surveillance data [48]. Older patients with Long COVID, especially those over 65, may be more likely to have died or been otherwise too functionally impaired to participate, contributing to possible selection bias for healthier older adults in the current study.

The regression model results suggest that a higher level of education attainment is associated with recovery from COVID-19, adding to existing published evidence indicating education and other social determinants of health (SDOH) are associated with Long COVID risk [49]. Participants without a bachelor’s degree had approximately 53% greater unadjusted Long COVID risk than those with an advanced degree and 33% greater with control for comorbidities and infection severity among other variables. More education is associated with lower unemployment and substantially greater household income and wealth [50], factors which facilitate health insurance coverage and healthcare access. People with more education may be positioned to have greater capacity to utilize and benefit from socially-determined resources to aid recovery (for example, paid time off from work, childcare, or effective healthcare) [51, 52].

Long COVID has been associated with greater odds of unemployment and lower odds of full-time employment, effects that were in turn strongly associated with both education and gender but not other SDOH variables [47]. An analysis by the Federal Reserve found roughly 25% of people with Long COVID reported their symptoms affected their ability to work, and Long COVID was associated with 50% fewer hours worked [53]. Losing or lacking full-time employment due to Long COVID could affect health insurance coverage, potentially limiting access to treatment for Long COVID. Discrimination and stigma experienced by those with Long COVID may create barriers in healthcare, occupational, and institutional settings that are more difficult to circumvent for those with less education or other socially marginalized characteristics [14, 42]. Qualitative studies have identified provider bias and Long COVID related stigma as obstacles to being diagnosed with and treated for Long COVID [42, 54]. As the National Academy of Science, Engineering, and Medicine describes, “bias and stigma affect whether patients can receive a diagnosis and benefit from Long COVID-targeted healthcare,” noting that relevant factors include healthcare and health insurance access and provider willingness to give a diagnosis of Long COVID [14].

Beyond education, evidence of other SDOH-related disparities in the prevalence of Long COVID were identified in our sample. Though female gender was not statistically significant in our multivariate results, the lower bound of the 95% confidence interval for its effect estimate was 0.996, indicating it may be associated with Long COVID risk. Additionally, self-classification as American Indian or Alaska Native was associated with increased risk in unadjusted terms, suggesting a possible racial disparity. Both findings are consistent with existing research on SDOH and Long COVID risk [14].

These results add to literature indicating SDOH-mediated inequities exist with respect to recovery from COVID-19 and the development of Long COVID. Rigorous, intersectional investigation into the mechanisms of how education, employment, gender, race, and other SDOH variables affect COVID-19 recovery within multilayered, interlocking social structures is crucial to identify how to mitigate inequitable long-term health effects of the pandemic [14, 55–57].

### Strengths and limitations

The strengths of this analysis include the long follow-up time after confirmed diagnosis, the inclusion of a Without Long COVID comparison group, and the sub-grouping of Long COVID into those with Current Long COVID and Recovered Long COVID. Patient report of functional impairment associated with Long COVID provides data that are not easily captured in electronic health records. However, since these data are cross-sectional and were reported retrospectively, reverse causation and recall bias cannot be ruled out. The QoL impairments detected with PROMIS measures may have preceded COVID-19 infection and could be a cause of prolonged recovery, rather than an effect of Long COVID. Current cognitive impairment or other health impairments could influence one’s recall of past comorbidities, infection severity, and COVID-19 recovery. Furthermore, we were unable to account for the effect of repeated infections, which could be an unmeasured explanatory variable for the associations identified.

Another limitation is that given the low response rate (6.0%), this sample may not be representative of the larger pool of eligible patients. The survey data could not be linked to EHR data to compare the survey responders to non-responders in terms of demographics and clinical characteristics. The proportion with Long COVID was substantially higher in this sample than the prevalence of Long COVID estimated in population-based studies [32], which may be due to response bias. Patients who attribute their current health impairments to COVID-19 could have been more likely to provide consent and respond to the survey, increasing their proportion in the sample relative to those who do not attribute current health to COVID-19 or who do not have current impairments. The representativeness of the results is further limited because those who died in the interim time between SARS-CoV-2 infection and the study could not be included, so their experience of Long COVID – potentially more severe than those included in the study – was not captured. The analysis of social factors was limited to gender, education, race, and ethnicity, all of which were self-classified based on United States Census categories, which restricts the opportunity to investigate SDOH in a more detailed manner.

Of note, 77% of participants were first diagnosed with COVID-19 in 2020, when either wild-type or the alpha variant were prominent and before the wide dissemination of COVID-19 vaccines. While this feature of the sample precludes analyzing the effect of vaccination and reduces the generalizability of findings when applied to more recent variants, it furthers our understanding of Long COVID among people who were first infected early in the pandemic – roughly 94 million globally and 20 million in the United States by the end of 2020, according to WHO estimates [58]. This patient group may require additional clinical considerations compared to those who were first infected by later variants.

## Conclusion

Using validated measures of health, this cross-sectional study found a high prevalence of cognitive decline and diminished QoL at a median of 2 years after SARS-CoV-2 infection for people who reported they did not recover from COVID-19 illness within 3 months. Compared to the Current Long COVID subgroup, the Recovered Long COVID subgroup showed evidence of recovery in most health domains assessed. However, compared to the Without Long COVID group, significant increases were identified in fatigue, pain, and most notably in cognitive decline for the Recovered group. These findings have implications for the sustainability of participation in work, educational, and social activities for people with Long COVID, even after perceived recovery or during periods of remission. Future work could investigate SDOH-related inequities in recovery and the differential effects of SARS-CoV-2 variants and vaccination. Furthermore, prospective longitudinal studies combining subjective and objective health measures would be valuable for mapping the trajectories of physical, neurological, and mental health associated with Long COVID.

## Supplementary Information

Supplementary Material 1.

Supplementary Material 2.

## Data Availability

The datasets used and/or analyzed during the current study are available from the corresponding author on reasonable request.

## Abbreviations

aRR: Adjusted Relative Risk
CCI-12: Cognitive Change Index 12
CDC: Centers for Disease Control and Prevention
COVID-19: Coronavirus Disease 2019
CI: Confidence Interval
ICD-10-CM: International Classification of Diseases, 10th Revision, Clinical Modification
ME/CFS: Myalgic Encephalomyelitis/Chronic Fatigue Syndrome
PCC: Post COVID-19 Condition
PCR: Polymerase Chain Reaction
PROMIS: Patient Reported Outcome Measurement Information System
QoL: Quality of Life
REDCap: Research Electronic Data Capture
RR: Relative Risk
SARS-CoV-2: Severe Acute Respiratory Syndrome Coronavirus 2
SDOH: Social Determinants of Health
WHO: World Health Organization
