# Supplementary material for: Long COVID and recovery from Long COVID: quality of life impairments and subjective cognitive decline at a median of 2 years after initial infection
Source: BMC Infect Dis. 2024 Nov 5;24:1241. doi: 10.1186/s12879-024-10158-w (PMC11536968; doi:10.1186/s12879-024-10158-w)
Supplement: Supplementary file 1 — Supplementary Material 1. [file 12879_2024_10158_MOESM1_ESM.docx]

## Supplemental Information

#### Long COVID Definitions

The survey included a question asking: “If you know, or believe, that you had COVID-19: have you recovered to your usual state of health?” Those who answered “Yes” were asked, “How long did it take for you to recover?” and were able to select the number of days and/or months.

All participants were at least 4 months from initial COVID infection when they completed surveys. Thus any participant answering “No” to the initial question about recovery was considered to have Long COVID, since they had not recovered within 3 months (n=181; “Current Long COVID”). Participants who answered “Yes” to the initial recovery question, but who reported a recovery time of >3 months were also considered part of the Long COVID group (n=34; “Recovered Long COVID”). Participants who answered “Yes” to the initial recovery question and who reported a recovery time of ≤3 months were defined as Without Long COVID (n=220).

#### Comparing the Current Long COVID Group to Recovered Long COVID

Descriptive statistics comparing the Current Long COVID group to the Recovered Long COVID group are shown in Table S2. Generally, there were few differences in the distributions of descriptive variables between these two groups, other than in current COVID symptom count. Some qualitative differences, that could be significant with greater sample size, were observed. The Recovered Long COVID group had a higher proportion of females (79.4%) than the Current Long COVID group (63.0%; *P*=0.08). While the Current Long COVID group had a larger proportion of those with no bachelor’s degree (40.9%) than those with an advanced degree (21.0%; *P*=0.23), the proportions were roughly equal in the Recovered Long COVID group (32.4% and 29.4%, respectively). The overall education distribution was not different between groups (*P=*0.49).

#### Identifying COVID Patients with Laboratory Testing and Diagnostic Codes

Patients could be included in the study with either a positive SARS-CoV-2 PCR test result or by a clinical diagnosis of COVID-19. A clinical diagnosis was identified by an International Classification of Diseases, 10th Revision, Clinical Modification (ICD-10-CM) diagnostic code of B97.29 (“Other coronavirus as the cause of diseases classified elsewhere”), and/or U07.1 (“COVID-19”). Those with a diagnostic code could have conducted testing outside of the university health system (e.g. with an at-home rapid test or a test administered at a local pharmacy). A higher proportion of those identified by diagnostic code: were Hispanic (*p*<0.01); had no bachelor’s degree, compared to an advanced degree (*p*<0.01); or were hospitalized for COVID-19 (*P*=0.02). See Table S3 for a comparison of demographics and clinical characteristics for the two identification methods. A sensitivity analysis was conducted by including the identification method in the regression analysis (see “Sensitivity Analyses” section below).

#### COVID-19 Symptom Counts

The number of symptoms experienced at the time of survey and during the acute phase of COVID-19 were totaled for each patient. The possible symptoms were the 11 core symptoms of COVID-19 published by the Centers for Disease Control and Prevention.(29) These symptoms are: fever or chills, cough, shortness of breath, fatigue, muscle or body aches, headache, new loss of taste or smell, sore throat, congestion or runny nose, nausea or vomiting, and diarrhea.

#### Sensitivity Analyses

*PROMIS-29 and CCI-12*

Missing values on the PROMIS questionnaires and the CCI-12 were imputed to bias the Without Long COVID group toward greater impairment and to bias the Long COVID group toward less impairment. For the Without Long COVID group, missing values were imputed with 5, indicating the maximum impairment on an item. Missing values for the Long COVID group were imputed with 1, indicating no impairment.

All significant findings presented in Table 2 and Figure 2 of the main text remained significant in this sensitivity analysis.

*COVID-19 Identification Method*

The method of infection confirmation was added to the multivariate regression model in the main analysis. Its addition did not affect the significance or point estimates of the other variables. The point estimate for the multivariate RR of Long COVID for those identified by PCR, compared to those identified with diagnostic code, was 0.99 (95% CI=0.80, 1.23).

*Time Since Index Diagnosis for Current Long COVID Group*

To examine if functional impairment burden differed within the Current Long COVID group by length of time since index diagnosis, we dichotomized time since diagnosis into those with 2 or more years since diagnosis and those with less than 2 years. These groups were compared in terms of the prevalence of moderate-to-severe health impairments (Table S4). We did not find any differences.

*Hospitalization*

Hospitalization due to COVID-19 was excluded from the regression analysis because 13.1% (n=57) of the sample reported a hospitalization between the time of SARS-CoV-2 infection and survey but did not give a reason for their hospitalization. Thus their COVID-19 hospitalization status could not be determined. A sensitivity analysis was conducted by separately adding two versions of the hospitalization variable to the final multivariable model from Table 3 in the primary analysis. In Sensitivity Model 1, the observations with missing values for hospitalization were removed from the analysis, giving a sample size of n=369. In Sensitivity Model 2, the observations were treated as a separate category in the model, representing a “Possible COVID-19 Hospitalization”.

The results of these two models are shown in Table S5 compared to the results of the primary analysis model. The point estimates were generally similar across the three models, though the patterns of statistical significance were not identical. A loss of statistical power was seen in Sensitivity Model 1, which had a smaller sample because the missing observations were excluded. This model had similar point estimates but wider confidence intervals compared to the model with hospitalization variable excluded entirely (Table 3 of main paper). The estimates and confidence intervals in Sensitivity Model 2 were similar to those computed in the main analysis.

#### TABLE S1. Missing Data Proportions by Variable

| Item | Missing | |
| --- | --- | --- |
|  | n | % |
| Age | 3 | 0.69 |
| Gender | 3 | 0.46 |
| Education | 4 | 0.92 |
| Race | 13 | 2.99 |
| Ethnicity | 11 | 2.53 |
| Hospitalization | 57 | 13.1 |
| Physical Function 1 | 2 | 0.46 |
| Physical Function 2 | 1 | 0.23 |
| Physical Function 3 | 6 | 1.38 |
| Physical Function 4 | 2 | 0.46 |
| Anxiety 1 | 6 | 1.38 |
| Anxiety 2 | 2 | 0.46 |
| Anxiety 3 | 3 | 0.69 |
| Anxiety 4 | 1 | 0.23 |
| Depression 1 | 3 | 0.69 |
| Depression 2 | 2 | 0.46 |
| Depression 3 | 3 | 0.69 |
| Depression 4 | 3 | 0.69 |
| Fatigue 1 | 3 | 0.69 |
| Fatigue 2 | 8 | 1.84 |
| Fatigue 3 | 3 | 0.69 |
| Fatigue 4 | 4 | 0.92 |
| Sleep 1 | 1 | 0.23 |
| Sleep 2 | 2 | 0.46 |
| Sleep 3 | 0 | – |
| Sleep 4 | 1 | 0.23 |
| Social Function 1 | 2 | 0.46 |
| Social Function 2 | 9 | 2.07 |
| Social Function 3 | 1 | 0.23 |
| Social Function 4 | 3 | 0.69 |
| Pain 1 | 3 | 0.69 |
| Pain 2 | 5 | 1.15 |
| Pain 3 | 4 | 0.92 |
| Pain 4 | 4 | 0.92 |
| CCI-12 1 | 8 | 1.84 |
| CCI-12 2 | 4 | 0.92 |
| CCI-12 3 | 8 | 1.84 |
| CCI-12 4 | 7 | 1.61 |
| CCI-12 5 | 8 | 1.84 |
| CCI-12 6 | 10 | 2.30 |
| CCI-12 7 | 7 | 1.61 |
| CCI-12 8 | 5 | 1.15 |
| CCI-12 9 | 5 | 1.15 |
| CCI-12 10 | 8 | 1.84 |
| CCI-12 11 | 17 | 3.91 |
| CCI-12 12 | 19 | 4.37 |

#### TABLE S2. Descriptive Statistics Comparing Current Long COVID to Recovered Long COVID

|  | **Total** | **Current** | **Recovered** |  |
| --- | --- | --- | --- | --- |
|  | **(N=215)** | **(N=181)** | **(N=34)** | **p-value*** |
| **Age** |  |  |  | 0.60 |
| 20-34 | 24 (11.2%) | 22 (12.2%) | 2 (5.9%) |  |
| 35-49 | 53 (24.7%) | 43 (23.8%) | 10 (29.4%) |  |
| 50-64 | 81 (37.7%) | 70 (38.7%) | 11 (32.4%) |  |
| 65+ | 54 (25.1%) | 44 (24.3%) | 10 (29.4%) |  |
| **Gender** |  |  |  | 0.08** |
| Male | 71 (33.0%) | 64 (35.4%) | 7 (20.6%) |  |
| Female | 141 (65.6%) | 114 (63.0%) | 27 (79.4%) |  |
| Other | 2 (0.9%) | 2 (1.1%) | 0 (0%) |  |
| **Education** |  |  |  | 0.49 |
| Advanced Degree | 48 (22.3%) | 38 (21.0%) | 10 (29.4%) |  |
| Bachelors | 79 (36.7%) | 66 (36.5%) | 13 (38.2%) |  |
| No bachelor’s | 85 (39.5%) | 74 (40.9%) | 11 (32.4%) |  |
| **Race** |  |  |  | 0.39 |
| White | 163 (75.8%) | 139 (76.8%) | 24 (70.6%) |  |
| Asian | 14 (6.5%) | 11 (6.1%) | 3 (8.8%) |  |
| Black | 13 (6.0%) | 11 (6.1%) | 2 (5.9%) |  |
| American Indian-Alaska Native | 6 (2.8%) | 5 (2.8%) | 1 (2.9%) |  |
| Other | 12 (5.6%) | 11 (6.1%) | 1 (2.9%) |  |
| **Hispanic Ethnicity** |  |  |  | 0.81 |
| Not Hispanic | 187 (87.0%) | 156 (86.2%) | 31 (91.2%) |  |
| Hispanic | 22 (10.2%) | 19 (10.5%) | 3 (8.8%) |  |
| **COVID Hospitalization** | 15 (7.0%) | 12 (6.6%) | 3 (8.8%) | 0.44 |
| **Comorbidities** |  |  |  |  |
| Cardiovascular (Non-Hypertension) | 25 (11.6%) | 24 (13.3%) | 1 (2.9%) | 0.20 |
| Hypertension | 47 (21.9%) | 38 (21.0%) | 9 (26.5%) | 0.48 |
| Diabetes | 19 (8.8%) | 18 (9.9%) | 1 (2.9%) | 0.32 |
| Respiratory | 37 (17.2%) | 30 (16.6%) | 7 (20.6%) | 0.62 |
| Clotting | 11 (5.1%) | 11 (6.1%) | 0 (0%) | 0.22 |
| Auto-immune | 22 (10.2%) | 20 (11.0%) | 2 (5.9%) | 0.54 |
| **Comorbidity Count** |  |  |  | 0.56 |
| 0 | 117 (54.4%) | 98 (54.1%) | 19 (55.9%) |  |
| 1 | 50 (23.3%) | 40 (22.1%) | 10 (29.4%) |  |
| 2 | 29 (13.5%) | 25 (13.8%) | 4 (11.8%) |  |
| 3 or more | 19 (8.8%) | 18 (9.9%) | 1 (2.9%) |  |
| **Acute Symptom Total;** Mean (SD)^†^ | 6.40 (2.66) | 6.35 (2.78) | 6.62 (1.97) | 0.51 |
| **Current Symptom Total;** Mean (SD) | 1.76 (2.07) | 1.98 (2.11) | 0.59 (1.37) | **<0.01** |
| **Time Since Infection (mos);** Median (IQR) | 22.6  (19.3, 26.8) | 24.1  (19.7, 26.8) | 24.2  (19.8, 26.9) | 0.39 |
| * Significance testing conducted with ­*t*-tests for continuous variables and Chi-squared tests for categorical. ** Results shown for pairwise comparison of Males and Females. ^†^ Symptom totals (both during the acute phase of COVID-19 and current) are counted out of 11 core symptoms defined by the Centers for Disease Control and Prevention: fever or chills, cough, shortness of breath, fatigue, muscle or body aches, headache, new loss of taste or smell, sore throat, congestion or runny nose, nausea or vomiting, and diarrhea. | | | | |

#### TABLE S3. Descriptive Statistics Comparing COVID-19 Patients Identified via Laboratory Test to Patients Identified via Diagnostic Code.

|  | **Total (N=435)** | **ICD (N=315)** | **PCR (N=120)** | **p-value*** |
| --- | --- | --- | --- | --- |
| **Age** |  |  |  | 0.64 |
| 20-34 | 61 (14.0%) | 45 (14.3%) | 16 (13.3%) |  |
| 35-49 | 105 (24.1%) | 74 (23.5%) | 31 (25.8%) |  |
| 50-64 | 148 (34.0%) | 105 (33.3%) | 43 (35.8%) |  |
| 65+ | 118 (27.1%) | 91 (28.9%) | 27 (22.5%) |  |
| **Gender** |  |  |  | 0.11** |
| Male | 166 (38.2%) | 128 (40.6%) | 38 (31.7%) |  |
| Female | 264 (60.7%) | 185 (58.7%) | 79 (65.8%) |  |
| Other | 3 (0.7%) | 1 (0.3%) | 2 (1.7%) |  |
| **Education** |  |  |  | **<0.01** |
| Advanced Degree | 123 (28.3%) | 78 (24.8%) | 45 (37.5%) |  |
| Bachelors | 166 (38.2%) | 119 (37.8%) | 47 (39.2%) |  |
| No bachelor’s | 142 (32.6%) | 114 (36.2%) | 28 (23.3%) |  |
| **Race** |  |  |  | 0.32 |
| White | 340 (78.2%) | 238 (75.6%) | 102 (85.0%) |  |
| Asian | 27 (6.2%) | 21 (6.7%) | 6 (5.0%) |  |
| Black | 27 (6.2%) | 21 (6.7%) | 6 (5.0%) |  |
| American Indian-Alaska Native | 8 (1.8%) | 8 (2.5%) | 0 (0%) |  |
| Other | 20 (4.6%) | 14 (4.4%) | 6 (5.0%) |  |
| **Hispanic Ethnicity** |  |  |  | **<0.01** |
| Not Hispanic | 385 (88.5%) | 272 (86.3%) | 113 (94.2%) |  |
| Hispanic | 39 (9.0%) | 36 (11.4%) | 3 (2.5%) |  |
| **Hospitalization** | 19 (4.4%) | 18 (5.7%) | 1 (0.8%) | **0.02** |
| **Comorbidities** |  |  |  |  |
| Cardiovascular (Non-Hypertension) | 39 (9.0%) | 30 (9.5%) | 9 (7.5%) | 0.51 |
| Hypertension | 86 (19.8%) | 62 (19.7%) | 24 (20.0%) | 0.94 |
| Diabetes | 30 (6.9%) | 25 (7.9%) | 5 (4.2%) | 0.17 |
| Respiratory | 59 (13.6%) | 47 (14.9%) | 12 (10.0%) | 0.18 |
| Clotting | 18 (4.1%) | 14 (4.4%) | 4 (3.3%) | 0.79 |
| Auto-immune | 41 (9.4%) | 32 (10.2%) | 9 (7.5%) | 0.40 |
| **Comorbidity Count** |  |  |  | 0.47 |
| 0 | 253 (58.2%) | 177 (56.2%) | 76 (63.3%) |  |
| 1 | 106 (24.4%) | 78 (24.8%) | 28 (23.3%) |  |
| 2 | 50 (11.5%) | 40 (12.7%) | 10 (8.3%) |  |
| 3 or more | 26 (6.0%) | 20 (6.3%) | 6 (5.0%) |  |
| **Acute Symptom Total;** Mean (SD)^†^ | 5.34 (2.83) | 5.39 (2.84) | 5.23 (2.80) | 0.61 |
| **Current Symptom Total;** Mean (SD) | 1.04 (1.73) | 1.00 (1.74) | 1.16 (1.71) | 0.38 |
| **Recovery Time (mos);** Median (IQR)^‡^ | 0.46  (0.20-1.97) | 0.46  (0.21-1.97) | 0.35  (0.16-0.69) | 0.06 |
| **Time Since Infection (mos);** Median (IQR) | 23.3  (18.6-26.7) | 21.6  (17.0-26.1) | 26.9  (24.4-27.9) | **<0.01** |
| **Self-Reported Long COVID** |  |  |  | 0.62 |
| Long COVID | 215 (49.4%) | 158 (50.2%) | 57 (47.5%) |  |
| Without Long COVID | 220 (50.6%) | 157 (49.8%) | 63 (52.5%) |  |
| * Significance testing conducted with ­*t*-tests for continuous variables and Chi-squared tests for categorical. ** Results shown for pairwise comparison of Males and Females. ^†^ Symptom totals (both during the acute phase of COVID-19 and current) are counted out of 11 core symptoms defined by the Centers for Disease Control and Prevention: fever or chills, cough, shortness of breath, fatigue, muscle or body aches, headache, new loss of taste or smell, sore throat, congestion or runny nose, nausea or vomiting, and diarrhea. ^‡^ Applies to patients reporting recovery to pre-COVID-19 baseline health at time of survey (n=254). | | | | |

#### Table S4. Moderate-to-Severe Impairment in Each Health Domain Assessed Stratified by Time Since Index Diagnosis Date for Current Long COVID Group

|  | **Total (N=181)** | **2 or more years**  **since index (N=91)** | **Less than 2 years since index (N=90)** | **p-value*** |
| --- | --- | --- | --- | --- |
| **Fatigue** | 81 (44.8%) | 42 (46.2%) | 39 (43.3%) | 0.64 |
| **Sleep Disturbance** | 38 (21.0%) | 18 (19.8%) | 20 (22.2%) | 0.79 |
| **Pain** | 70 (38.7%) | 35 (38.5%) | 35 (38.9%) | 0.88 |
| **Physical Function** | 72 (39.8%) | 41 (45.1%) | 31 (34.4%) | 0.11 |
| **Social Function** | 46 (25.4%) | 23 (25.3%) | 23 (25.6%) | 0.93 |
| **Anxiety** | 53 (29.3%) | 31 (34.1%) | 22 (24.4%) | 0.15 |
| **Depression** | 36 (19.9%) | 19 (20.9%) | 17 (18.9%) | 0.73 |
| **Cognitive Decline** | 134 (74.0%) | 67 (73.6%) | 67 (74.4%) | 0.86 |
| **Length of Illness (mos)** | 22.7 (5.63) | 27.0 (1.47) | 18.4 (4.95) | – |
| ***** Significance testing conducted with chi-squared tests. | | | | |

#### TABLE S5. Comparison of the Multivariable Regression Model from the Main Analysis to Two Sensitivity Models including COVID-19 hospitalization

|  | **Main Analysis Model (n=424)** | | **Sensitivity Model 1 (n=369)*** | | **Sensitivity Model 2 (n=424)** | |
| --- | --- | --- | --- | --- | --- | --- |
|  | Adj. RR (95% CI) | p-value | Adj. RR (95% CI) | p-value | Adj. RR (95% CI) | p-value |
| **Age** |  |  |  |  |  |  |
| 20 – 34 | Reference | – | Reference | – | Reference | – |
| 35 – 49 | 1.47 (1.01, 2.13) | **0.05** | 1.38 (0.92, 2.08) | 0.11 | 1.42 (0.97, 2.08) | 0.07 |
| 50 – 64 | 1.52 (1.06, 2.18) | **0.03** | 1.44 (0.96, 2.15) | 0.07 | 1.49 (1.04, 2.15) | **0.03** |
| 65+ | 1.48 (1.01, 2.18) | **0.05** | 1.31 (0.86, 2.02) | 0.21 | 1.45 (0.98, 2.14) | 0.06 |
| **Gender** |  |  |  |  |  |  |
| Male | Reference | – | Reference | – | Reference | – |
| Female | 1.23 (1.00, 1.51) | 0.06 | 1.12 (0.88, 1.41) | 0.37 | 1.24 (1.00, 1.53) | **0.05** |
| **Education** |  |  |  |  |  |  |
| Advanced Degree | Reference | – | Reference | – | Reference | – |
| Bachelor’s Degree | 1.12 (0.87, 1.46) | 0.38 | 1.15 (0.85, 1.56) | 0.36 | 1.11 (0.86, 1.44) | 0.41 |
| No bachelor’s Degree | 1.33 (1.03, 1.71) | **0.03** | 1.47 (1.09, 1.97) | **0.01** | 1.32 (1.03, 1.70) | **0.03** |
| **Race** |  |  |  |  |  |  |
| White | Reference | – | Reference | – | Reference | – |
| Asian | 1.12 (0.75, 1.67) | 0.59 | 1.09 (0.70, 1.70) | 0.69 | 1.15 (0.77, 1.71) | 0.49 |
| Black | 0.90 (0.62, 1.29) | 0.57 | 0.93 (0.65, 1.32) | 0.69 | 0.90 (0.62, 1.31) | 0.59 |
| American Indian/Alaska Native | 1.09 (0.67, 1.78) | 0.73 | 1.11 (0.64, 1.94) | 0.71 | 1.10 (0.69, 1.76) | 0.69 |
| Other | 1.06 (0.66, 1.71) | 0.81 | 1.01 (0.54, 1.90) | 0.98 | 1.06 (0.66, 1.71) | 0.81 |
| **Hospitalization** |  |  |  |  |  |  |
| No COVID-19 Hospitalization | – | – | Reference | – | Reference | – |
| COVID-19 Hosp. | – | – | 1.26 (0.94, 1.69) | 0.12 | 1.33 (1.00, 1.77) | **0.05** |
| Possible COVID-19 Hosp.** | – | – | – | – | 1.23 (1.00, 1.52) | 0.06 |
| **Pre-Infection Comorbidities** |  |  |  |  |  |  |
| No Comorbidities | Reference | – | Reference | – | Reference | – |
| 1 | 0.93 (0.74, 1.18) | 0.56 | 0.94 (0.72, 1.23) | 0.67 | 0.92 (0.73, 1.16) | 0.46 |
| 2 | 1.15 (0.89, 1.50) | 0.29 | 1.08 (0.80, 1.47) | 0.61 | 1.12 (0.87, 1.45) | 0.39 |
| 3 or more | 1.45 (1.11, 1.90) | **<0.01** | 1.65 (1.17, 2.34) | **<0.01** | 1.30 (0.96, 1.75) | 0.09 |
| **Total Number of Acute Symptoms** | 1.14 (1.10, 1.18) | **<0.01** | 1.14 (1.09, 1.18) | **<0.01** | 1.13 (1.09, 1.17) | **<0.01** |
| * Sensitivity Model 1 excluded n=57 whose hospitalization reason could not be determined. ** Sensitivity Model 2 included the n=57 missing as a separate category, representing a Possible COVID-19 hospitalization. The “Missing” categories for age (n=3), gender (n=2), and education (n=4), and the Other category for gender (n=3) were dropped from the analysis due to group size of <5. Not displayed: “Missing” category for race (n=12). *RR* Relative Risk, *CI* Confidence Interval | | | | | | |
