# Supplementary material for: Long COVID and recovery from Long COVID: quality of life impairments and subjective cognitive decline at a median of 2 years after initial infection
Source: BMC Infect Dis. 2024 Nov 5;24:1241. doi: 10.1186/s12879-024-10158-w (PMC11536968; doi:10.1186/s12879-024-10158-w)
Supplement: Supplementary file 2 — Supplementary Material 2. [file 12879_2024_10158_MOESM2_ESM.pdf]

COVID-19 RELIEF Questionnaire

Please complete the following questions to the best of your ability.

COVID-19 TESTS

1. **Have you been tested for coronavirus or COVID-19 (report on PCR tests only and not home-based tests)?**

   Yes No Unsure

   If yes, have you ever had a test for:

   a. COVID-19 infection? Yes No (If yes, result: Positive Negative)
      If yes, date of first test: _______________

   b. COVID-19 immunity Yes No (If yes, result: Positive Negative)
      If yes, date of first test:________________

   c. How many times have you been tested? __________

      Date of first test: ______________ (month/year) Date of most recent test: ______________ (month/year)

2. **Have you had an overnight stay in a hospital since your first COVID-19 test (positive or negative). Please include hospitalizations for COVID-19 or for other reasons.**

   Yes No (if no, skip to Q #3)

   If yes – please describe each hospitalization after your first COVID-19 PCR test:

   a. How many nights were you in the hospital (first hospitalization after COVID-19 diagnosis)?
      i. Date arrived at hospital: __________________
      ii. Date discharged from hospital: _____________
      iii. Hospital:

         ___ Harborview Medical Center
         ___ UW Medical Center (UW Medical Center – Montlake)
         ___ Northwest Hospital (UW Medical Center – Northwest)
         ___ Other:____

      iv. Reason for hospitalization: Suspected or diagnosed COVID-19
          Other:_______________________________

      v. While in the hospital, did you spend any time in the ICU? Yes No Unsure
      vi. While in the hospital, were you ventilated? Yes No Unsure

3. **Have you seen a doctor or other health care provider at an office visit (including telehealth or phone visit) about health care concerns due to COVID-19 since your first COVID-19 PCR test?**

    Yes   No

    If yes:

    a. How many office visits have you had? ____________________
    b. Date of first visit: _________________
    c. Date of most recent visit: ____________
    d. Name of physician: ____
    e. Location of outpatient encounter:
        ___ Harborview Medical Center
        ___ UW Medical Center at Montlake
        ___ UW Medical Center at Northgate
        ___ UW Neighborhood Clinics
        ___ Other:____

4. **If you know, or believe, that you had COVID-19: have you recovered to your usual state of health?**

    Yes   No

    a. ***If yes***: how long did it take for you to recover? ____________ days

PREVIOUS AND CURRENT COVID-19 SYMPTOMS

We are now going to ask you about the symptoms you may have had during your initial phase of the illness (after being diagnosed) and how you are feeling now (any persisting or new symptoms). It is possible that these symptoms were severe or that you had mild or no symptoms at all. Do your best to remember – we are providing a list of different symptoms we'd like you to tell us about.

5. COVID PATIENTS: Please tell us about the symptoms you had at the time of your COVID-19 infection and if you currently have them.

CONTROLS (no reported COVID): Think about the way you were feeling on or around DATE OF MATCHED CASE. Please tell us if you had any of these symptoms during that time period.

	A. During your COVID-19 illness, did you have worsening of this symptom compared to your usual state of health?	B. When the symptom was at its worst, how much did it bother you, on a scale of 1 to 10?	C. How long did the symptom last?	D. Do you currently have this symptom?	E. How much does it bother you now on a scale of 1 to 10?
Fever > 100.4 F	Yes / No				
Subjective fever (felt feverish)	Yes / No				
Chills or shivering	Yes / No				
Difficulty breathing	Yes / No				
Shortness of breath / faster breathing	Yes / No				
Chest congestion	Yes / No				
Chest pain	Yes / No				
Dry or hacking cough (new onset or worsening of chronic condition)	Yes / No				
Wet or loose cough	Yes / No				
Headache	Yes / No				
Muscle aches or pains (myalgia)	Yes / No				
Sore or painful throat	Yes / No				
Congested or stuffy nose	Yes / No				
Runny or dripping nose	Yes / No				
Nausea or vomiting	Yes / No				
Abdominal pain	Yes / No				
Diarrhea	Yes / No				
Fatigue (weak or tired)	Yes / No				
Loss of smell	Yes / No				
Loss of taste	Yes / No				
Other (describe):					
Overall, when these symptoms were at their worst, when you had these symptoms, how bad or bothersome were they? (Patient Global Rating of Flu Severity Instrument)<br>Mild Moderate Severe Very severe					

5. COVID PATIENTS: Please tell us about the symptoms you had at the time of your COVID-19 infection and if you currently have them.<br><br>CONTROLS (no reported COVID): Think about the way you were feeling on or around DATE OF MATCHED CASE. Please tell us if you had any of these symptoms during that time period.					
	**A. During your COVID-19 illness, did you have worsening of this symptom compared to your usual state of health?**	**B. When the symptom was at its worst, how much did it bother you, on a scale of 1 to 10?**	**C. How long did the symptom last?**	**D. Do you currently have this symptom?**	**E. How much does it bother you now on a scale of 1 to 10?**
Overall, when these symptoms were at their worst, did they interfere with your daily activities? (Patient Global Assessment of Interference with Daily Activities)<br>Not at all A little bit Somewhat Quite a bit Very much					

6. Has a doctor or other health care professional told you that you have/had:				
	A. Yes/No	B. Date (month/year)	C. Did it first occur before or after your COVID-19 symptoms	D. Are you taking medication for the condition?
Heart attack, myocardial infarction, MI	Yes / No		Yes / No	Yes / No
Angina or angina pectoris	Yes / No		Yes / No	Yes / No
Stroke	Yes / No		Yes / No	Yes / No
Mini-stroke, transient ischemic attack or TIA	Yes / No		Yes / No	Yes / No
Diabetes mellitus	Yes / No		Yes / No	Yes / No
Hypertension or high blood pressure	Yes / No		Yes / No	Yes / No
Congestive Heart Failure (CHF) or Heart Failure	Yes / No		Yes / No	Yes / No
Other cardiovascular disease (Specify):	Yes/ No		Yes/ No	Yes/ No
Deep vein thrombosis or blood clots in your legs	Yes / No		Yes / No	Yes / No
Other blood clots or clotting disorder, Specify):	Yes/ No		Yes/ No	Yes/ No
Chronic Renal Disease	Yes / No		Yes / No	Yes / No
Chronic Liver Disease	Yes / No		Yes / No	Yes / No
Asthma	Yes / No		Yes / No	Yes / No
Emphysema	Yes / No		Yes / No	Yes / No
COPD or chronic obstructive pulmonary disease	Yes / No		Yes / No	Yes / No
Other lung disease (Specify):	Yes / No		Yes / No	Yes / No
Lupus erythmetosis or lupus	Yes / No		Yes / No	Yes / No
Rheumatoid arthritis	Yes / No		Yes / No	Yes / No
Autoimmune liver disorder	Yes / No		Yes / No	Yes / No
Other autoimmune disorder (Specify):	Yes / No		Yes / No	Yes / No
Other Chronic Disease (Specify):	Yes / No		Yes / No	Yes / No
Other Chronic Disease (Specify):	Yes / No		Yes / No	Yes / No
Other Chronic Disease (Specify):	Yes/ No		Yes/ No	Yes/ No

We are now going to focus on some specific health concerns that you may or may not have experienced in the last month.

POST-COVID FATIGUE

7. Below is a list of symptoms many individuals experience. Please tell us whether or not you have any of the symptoms listed below. FOR EACH SYMPTOM, PLEASE CIRCLE THE APPROPRIATE ANSWERS IN THE GRID BELOW.

	During the past month, have you had this symptom? Please circle 1 or 2. If 1 is selected, then go to Column A		COLUMN A<br>Prior to this past month, for how long had you experienced this symptom?		COLUMN B<br>Did you have this symptom BEFORE you tested positive for COVID-19?	
	YES	NO	UNDER 3 MONTHS	3 MONTHS OR LONGER	YES	NO
Fatigue, tiredness, or exhaustion	1	2	1	2	1	2
Post-activity or post-exertional fatigue	1	2	1	2	1	2
Muscle aches/muscle pains	1	2	1	2	1	2
Pain in joints	1	2	1	2	1	2
Unrefreshing sleep	1	2	1	2	1	2
Problems getting to sleep, sleeping through the night, or waking up on time	1	2	1	2	1	2
Forgetfulness/memory problems that caused you to substantially cut back on your activities	1	2	1	2	1	2
Difficulty thinking or concentrating that caused you to substantially cut back on your activities	1	2	1	2	1	2
Dizziness or fainting	1	2	1	2	1	2

8. For each symptom you noted in question 7, please fill in the grid to describe the frequency and the severity of the symptoms. FOR EACH SYMPTOM, PLEASE CIRCLE THE APPROPRIATE ANSWERS IN THE GRID BELOW.

SYMPTOMS	DURING THE PAST MONTH, HOW OFTEN HAVE YOU HAD THIS SYMPTOM?					DURING THE PAST MONTH, HOW BAD WAS THIS SYMPTOM?				
	A LITTLE OF THE TIME	SOME OF THE TIME	A GOOD BIT OF THE TIME	MOST OF THE TIME	ALL OF THE TIME	VERY MILD	MILD	MODE RATE	SEVERE	VERY SEVE RE
Fatigue, tiredness or exhaustion	1	2	3	4	5	1	2	3	4	5
Post-activity or post-exertional fatigue										
Muscle aches/muscle pains	1	2	3	4	5	1	2	3	4	5
Pain in joints	1	2	3	4	5	1	2	3	4	5
Unrefreshing sleep	1	2	3	4	5	1	2	3	4	5
Problems getting to sleep, sleeping through the night, or waking up on time	1	2	3	4	5	1	2	3	4	5
Forgetfulness/memory problems that caused you to substantially cut back on your activities	1	2	3	4	5	1	2	3	4	5
Difficulty thinking or concentrating that caused you to substantially cut back on your activities	1	2	3	4	5	1	2	3	4	5

Dizziness or fainting	1	2	3	4	5	1	2	3	4	5

9. For Fatigue symptom you noted in question 5, please answer the following questions.

a. When this fatigue, tiredness, or exhaustion began, would you say that it came on all of a sudden, or slowly over time?

☐1 All of sudden
☐2 Slowly over time
☐6 Not applicable
☐8 Don't know

b. In what month and year did your fatiguing illness begin?
Month_____ Year_____

c. When you are fatigued, does rest make your fatigue better?
☐1 Yes, a lot
☐2 Yes, a little
☐3 No, not very much
☐6 Not applicable
☐8 Don't know

d. When you are fatigued, has this fatigue substantially limited your ability to occupational, educational, social, or personal activities?
☐1 Yes
☐2 No
☐6 Not applicable
☐8 Don't know

10. For the symptoms noted in question 7.
Do any of them get worse for at least 24 hours after you engage in activities (physical or mental) that you were used to doing .... with no problems?
☐1 Yes
☐2 No
☐6 Not applicable
☐8 Don't know

PROMIS – 29 PROFILE

11. Please tell us more about your current health. This this does not need to be from COVID but how you are generally doing now. Please respond to each question or statement by marking one box for each,

Physical Function

	**Without any Difficulty**	**With a little difficulty**	**With some s difficulty**	**With much difficulty**	**Unable to do**
**a. Are you able to do chores such as vacumming or yard work?**	5	4	3	2	1
**b. Are you able to up and down stairs at a normal pace?**	5	4	3	2	1

**c. Are you able to go for a walk of at least 15 minutes?**	5	4	3	2	1
**d. Are you able to run errands and shop?**	5	4	3	2	1

Anxiety

**In the past 7 days….**	**Never**	**Rarely**	**Sometimes**	**Often**	**Always**
**a. I felt fearful……**	1	2	3	4	5
**b. I found it hard to focus on anything other than my anxiety…….**	1	2	3	4	5
**c. My worries overwhelmed me……**	1	2	3	4	5
**d. I felt uneasy……**	1	2	3	4	5

Depression

**In the past 7 days….**	**Never**	**Rarely**	**Sometimes**	**Often**	**Always**
**a. I felt worthless……**	1	2	3	4	5
**b. I felt helpless…….**	1	2	3	4	5
**c. I felt depressed……**	1	2	3	4	5
**d. I felt hopeless……** s	1	2	3	4	5

Fatigue

**In the past 7 days….**	**Not at all**	**A little bit**	**Somewhat**	**Quite a bit**	**Very much**
**a. I felt fatigued**	1	2	3	4	5
**b. I have trouble <u>starting</u> things because I am tired**	1	2	3	4	5
**c. How run down did you feel on average?**	1	2	3	4	5
**d. How fatigued were you on average?**	1	2	3	4	5

Sleep Disturbance

**In the past 7 days….**	**Very poor**	**Poor**	**Fair**	**Good**	**Very good**

**a. My sleep quality was…..**	5	4	3	2	1

In the past 7 days….

	Not at all	A little bit	Somewhat	Quite a bit	Very much
**a. My sleep was refreshing…..**	5	4	3	2	1
**b. I had a problem with my sleep….**	5	4	3	2	1
**c. I had difficulty falling asleep…..**	5	4	3	2	1

Ability to Participate in Social Roles and Activities

	Not at all	A little bit	Somewhat	Quite a bit	Very much
**a. I have trouble doing all of my regular leisure activities with others**	1	2	3	4	5
**b. I have trouble doing all of the family activities that I want to do**	1	2	3	4	5
**c. I have trouble doing all of my usual work (include work at home)**	1	2	3	4	5
**d. I have trouble doing all of the activities with friends that I want to do**	1	2	3	4	5

Pain Interference

In the past 7 days….

	Not at all	A little bit	Somewhat	Quite a bit	Very much
**a. How much did pain interfere with your day-to-day activities?**	1	2	3	4	5
**b. How much did pain interfere with work around the home?**	1	2	3	4	5
**c. How much did pain interfere with your ability to participate in social activities?**	1	2	3	4	5
**d. How much did pain interfere with your household chores?**	1	2	3	4	5

Pain Intensity

In the past 7 days….

a. How would you rate your pain on average on a scale of 0 to 10?

No pain 0 1 2 3 4 5 6 7 8 9 10 **Worst pain imaginable**

MEMORY AND COGNITION

Please tell us about any changes that may have occurred in terms of your memory or related symptoms ("brain fog") since your COVID-19 positive test (or index date if negative control).

The following questions ask you to rate your memory compared to before your positive COVID-19 test. Each question uses a scale from 1 to 5, where 1 means no change in your memory since the test, 2 is minimal change, 3 is some change, 4 is moderate change, and 5 is much worse.

Since your positive COVID-19 Test, how would you rate your ability to:

	Change in Memory since COVID Test				
	None	Minimal	Some Change	Moderate Change	Much Worse
a. Recall information when you really try	1	2	3	4	5
b. Remember names and faces of new people that you meet	1	2	3	4	5
c. Remember things that have happened recently	1	2	3	4	5
d. Recall conversations a few days later	1	2	3	4	5
e. Remember where things are usually kept	1	2	3	4	5
f. Remember new information told to you	1	2	3	4	5
g. Remember where you placed familiar objects	1	2	3	4	5
h. Remember what you intended to do	1	2	3	4	5
i. Remember names of family members and friends	1	2	3	4	5
j. Remember without notes and reminders	1	2	3	4	5
k. Remember things compared to other people your age	1	2	3	4	5
l. How would people who know you rate your memory relative to 5 years ago?	1	2	3	4	5

How concerned are you about the changes you described above? Would you say you are:

1) Not at all concerned
2) Slightly concerned
3) Mildly concerned
4) Moderately concerned
5) Extremely concerned

12. Have you received the COVID-19 vaccination?

Yes No Unsure

a. If yes, which one:

Moderna
Pfizer
Johnson & Johnson
Astro Zeneca
Other: ________________________________
Unsure

b. Date of vaccination:

Dose 1	_____/_____/____	
Dose 2	____/_____/_____	OR have not received 2nd dose
Dose 3 or booster	_____/_____/____	OR have not received 3rd dose/booster

Please tell us about your yourself:

13. Do you currently use any tobacco products?

a. Cigarettes	Yes	No	if yes: cigarettes per day:__________
b. Pipes	Yes	No	if yes: number per day:__________
c. Cigars	Yes	No	if yes: number per day:__________
d. E-cigarettes	Yes	No	if yes: times per day:__________
e. Other: ____________________			

13. Do you currently drink alcohol (including beer, wine or hard liquor) on a regular basis (e.g 1 or more per week)?

f. Beer	Yes (if yes: bottles/cans per week:__________)	No
g. Wine	Yes (if yes: glasses per week___________)	No
h. Liquor	Yes (if yes: shots per week:__________)	No
i. Other alcoholic drinks	Yes (if yes: glasses per week:__________)	No

Height: ______/ ________
Feet Inches

Weight: ___________ pounds

Health Insurance (0lease check as many as apply)**:**

_____ None
_____ Medicare
_____ Medicaid
_____ Private Insurance
(specify):_____________________

Demographics:

What is your age: _______ years

What gender are you?
_____ Male
_____ Female
_____ Other:__________________________
_____ Prefer not to say

What is your race?
_____ White/Caucasian
_____ Black/African American
_____ Asian
_____ Native American/Pacific Islander
_____ Other:__________________________

Are you of Hispanic Ethnicity? Yes No Unsure Prefer not to say

What is the highest level of education that you have achieved?

_____ Less than a high school degree
_____ High School diploma
_____ Some college / Associates degree
_____ Bacholor's or other 4 year college degree
_____ Master's degree
_____ PhD degree
_____ Professional degree (MD, JD, DDS, etc).
_____ Prefer not to say

We are hoping to link additional information from your medical records from any hospitalizations or clinic visits at University of Washington Medicine. May we have your permission to link responses from this questionnaire to your medical record?

Yes No Unsure – please contact me for further information

We are planning to collect blood and saliva samples from some UW patients who gave tested positive for COVID-19. We will send a kit to your home that you will use to provide a very small amount of blood and a tube for collecting saliva. We will compensate those who send us samples with a $20 Amazon gift card. Would you be interested in participating in this study? If yes, we will send you more information about it.

Yes No Unsure

THANK YOU SO MUCH FOR COMPLETING THIS QUESTIONNAIRE. Your responses will be very valuable in helping us learn more about symptoms following a positive COVID-19 test. Please don't hesitate to contact us should you have additional questions at UWCOVIDRELIEF@uw.edu.
